# Supplementary material for: Stroboscopic operando spectroscopy of the dynamics in heterogeneous catalysis by event-averaging
Source: Nat Commun. 2021 Oct 21;12:6117. doi: 10.1038/s41467-021-26372-y (PMC8531341; doi:10.1038/s41467-021-26372-y)
Supplement: Supplementary file 1 — Supplementary Information [file 41467_2021_26372_MOESM1_ESM.pdf]

Supplementary information for:

Stroboscopic operando spectroscopy of the dynamics in heterogeneous catalysis by event-averaging

Jan Knudsen[1,2*], Tamires Gallo[1], Virgínia Boix[1], Marie Døvre Strømsheim[3], Giulio D'Acunto[1], Christopher Goodwin[4], Harald Wallander[1], Suyun Zhu[2], Markus Soldemo[4], Patrick Lömker[5], Filippo Cavalca[2], Mattia Scardamaglia[2], David Degerman[4], Anders Nilsson[4], Peter Amann[4], Andrey Shavorskiy[2], Joachim Schnadt[1,2]

[1]Division of Synchrotron Radiation Research, Department of Physics, Lund University, Sweden

[2]MAX IV Laboratory, Lund University, Sweden

[3] Department of Chemical Engineering, Norwegian University of Science and Technology (NTNU), Norway

[4]Department of Physics, Stockholm University, Sweden

[5]Deutsches Elektronen-Synchrotron DESY, Germany

*Corresponding author: jan.knudsen@sljus.lu.se

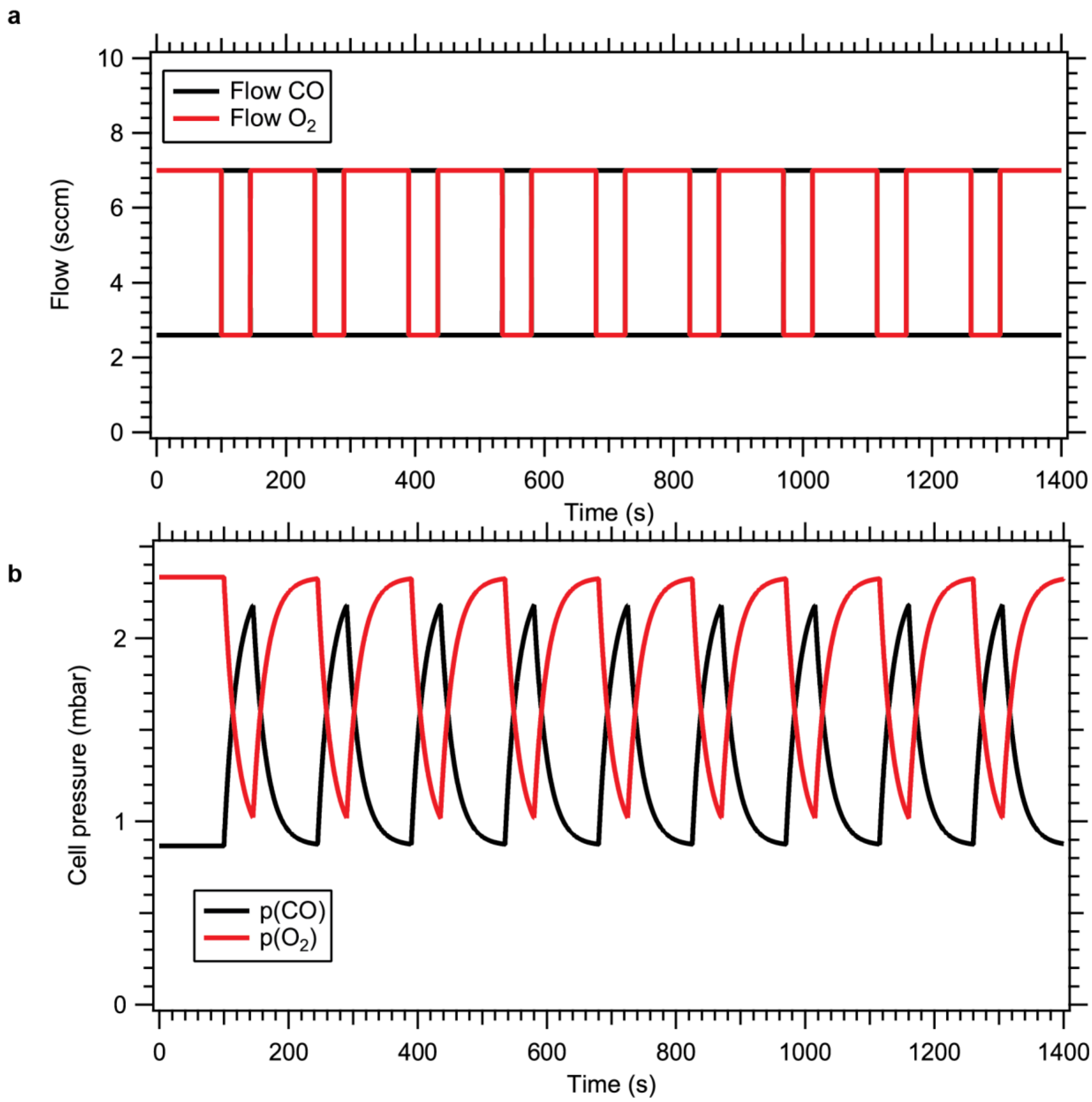

Supplementary Figure 1. **Flows and calculated pressures that are expected with no catalytic activity for the experiments plotted in figure 1 of the main article.** **a** Flow pattern used for the *O 1s*, *Pd* $3d_{5/2}$, and *C 1s* data shown in figure 1 and supplementary figures 6 and 7, respectively. **b** Simulated partial pressures assuming a 1 l cell, no conversion, and complete and instantaneous mixing in the reactor cell volume.

Even though the switching time of the mass flow controllers is rapid and below 300 ms it is important to realize that the gas composition in the cell is changed much slower due to the limited flow and relatively large volume of the cell. This figure simulates the time-evolution of CO and O_2 partial pressures in a gedankenexperiment with no conversion and demonstrates that the CO partial pressure would oscillate between 0.9 and 2.2 mbar as rising (45 s) and falling (100 s) exponential functions. For simplicity, the ~ 120 s time delay from the gas switching event until the gas composition started to change in the reactor cell is not included in the simulation. This delay is due to the gas travel time from the mass flow controllers to the cell volume.

Details of the simulation: The partial pressures of CO and O_2 are calculated using the following equations:

$$p_{O2}(0\ s) = \frac{Flow_{O2}(0\ s)}{Flow_{tot}(0\ s)} \cdot p_{tot} = \frac{7\ sccm}{9.6\ sccm} \cdot 3.2\ mbar = 2.3\ mbar \quad (1)$$

$$p_{CO}(0\ s) = \frac{Flow_{CO}(0\ s)}{Flow_{tot}(0\ s)} \cdot p_{tot} = \frac{2.6\ sccm}{9.6\ sccm} \cdot 3.2\ mbar = 0.87\ mbar \quad (2)$$

$$p_{O2}(t + dt) = \frac{V_{O2}(t+dt)}{V_{cell}} \cdot p_{tot} = \frac{p_{tot}}{V_{cell}} \cdot \left(\frac{p_{O2}(t)}{p_{tot}} \cdot V_{cell} + Flow_{O2}(t) \cdot dt - Flow_{tot} \frac{p_{O2}(t)}{p_{tot}} \cdot dt\right) \quad (3)$$

$$p_{CO}(t + dt) = \frac{V_{CO}(t+dt)}{V_{cell}} \cdot p_{tot} = \frac{p_{tot}}{V_{cell}} \cdot \left(\frac{p_{CO}(t)}{p_{tot}} \cdot V_{cell} + Flow_{CO}(t) \cdot dt - Flow_{tot} \frac{p_{CO}(t)}{p_{tot}} \cdot dt\right) \quad (4)$$

where V_{cell} is the volume of the cell (1000 ml), $Flow_{tot}$ is the total flow (9.6 sscm), p_{tot} the total pressure (3.2 mbar), and p_{O2}, p_{CO}, $Flow_{O2}$, and $Flow_{CO}$ are time dependent partial pressures and flows of O_2 and CO, respectively. When performing the simulation it is important to convert flows in sccm units to real flows in ml / s at the cell pressure of 3.2 mbar and all flows therefore need to be multiplied by a factor of (1000/3.2/60). In equation (3) and (4) the partial pressures of O_2 and CO are calculated from the volumetric compositional changes within the cell.

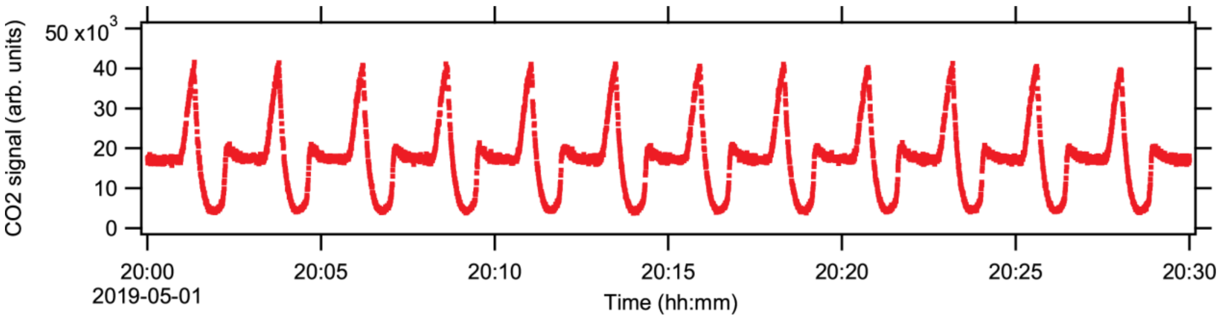

Supplementary Figure 2. **CO_2 signal that shows the oscillations induced by the gas-composition modulation recorded by a mass spectrometer that probes the gas flowing into the 0.3 mm nozzle of the electron analyser**. A fraction of the gas pumped through the nozzle was leaked into a dedicated mass spectrometer chamber equipped with a Hiden (HAL/3F PIC) mass spectrometer, to ensure rapid response to gas composition changes and a low baseline signal. The base pressure of the mass spectrometer chamber was ~ $1 \cdot 10^{-9}$ mbar while a pressure of ~ $5 \cdot 10^{-8}$ mbar was observed during the experiments.

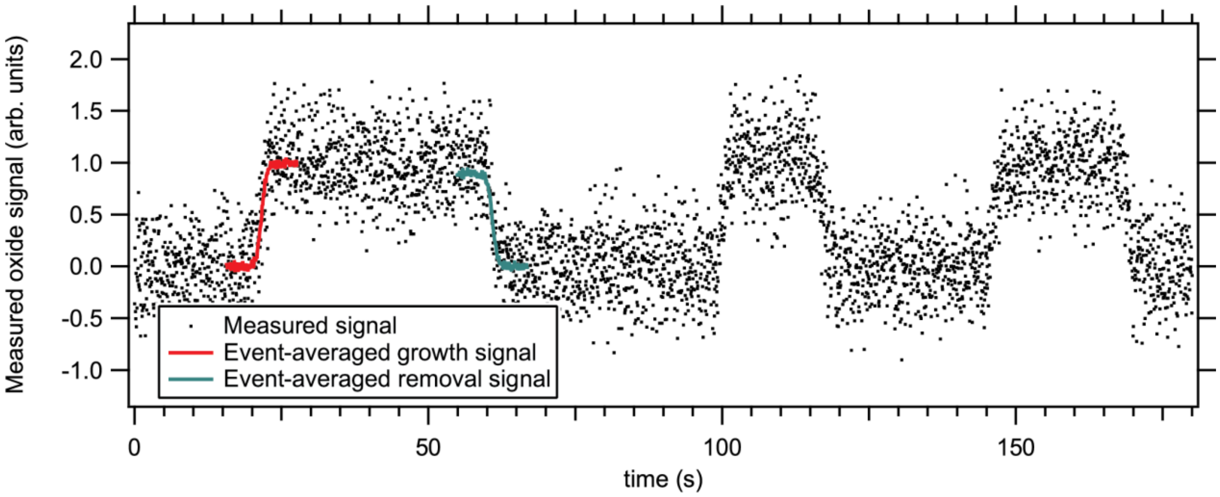

Supplementary Figure 3. **Simulation of how non-periodic reaction oscillation can be event-averaged.** In a gedankenexperiment a non-periodic and self-sustained reaction is observed by a surface sensitive signal, a gas phase signal, or both. In such a case it is impossible to event-average by an external signal as no external stimuli drives the oscillations. An example of such a situation can be found in figure 1 of ref. [1] where the inverse full width at half maximum ($FWHM^{-1}$) of the (h, k, l) = (1, 0, 2) diffraction peak is used to follow self-sustained smoothness oscillations of a Pd(001) surface while running the CO oxidation reaction. At the same time oscillations are observed in the measured CO_2 production.
Using a rising and falling edge algorithm in the gedanken- experiment one can detect the absolute time of each transition event. This is very similar to what is done with an oscilloscope. The only difference is that the data analysis is done after the experiment instead of on the fly as with the oscilloscope.

In the example used here 100 rising and falling edges, respectively, are event-averaged in a time window from -6 s to + 6 s around the absolute time of each transition. Clearly, the signal is improved substantially by event-averaging. In the example chosen here a 1D signal (for example $FWHM^{-1}$) is measured as function of time. The exact same methodology can, however, also be used for 2D(t) and 3D(t) signals. For example, we illustrate how a 2D spectral change can be used as an internal triggering signal in the article. An example of 3D(t) signal could be SXRD where (h, k, l, t) signals that in the future should be possible to record extremely fast with fast 2D detectors and fast spinning samples.

<u>Details of the simulation:</u> First non-periodic signal oscillating between 0 and 1 was generated using a Gauss error function (erf) together with random pulse widths and inter-pulse periods (between 15 and 40 s). Subsequently, Gaussian noise having a standard deviation of 0.3 was added. Finally, 100 rising and falling edges, respectively, were event-averaged.

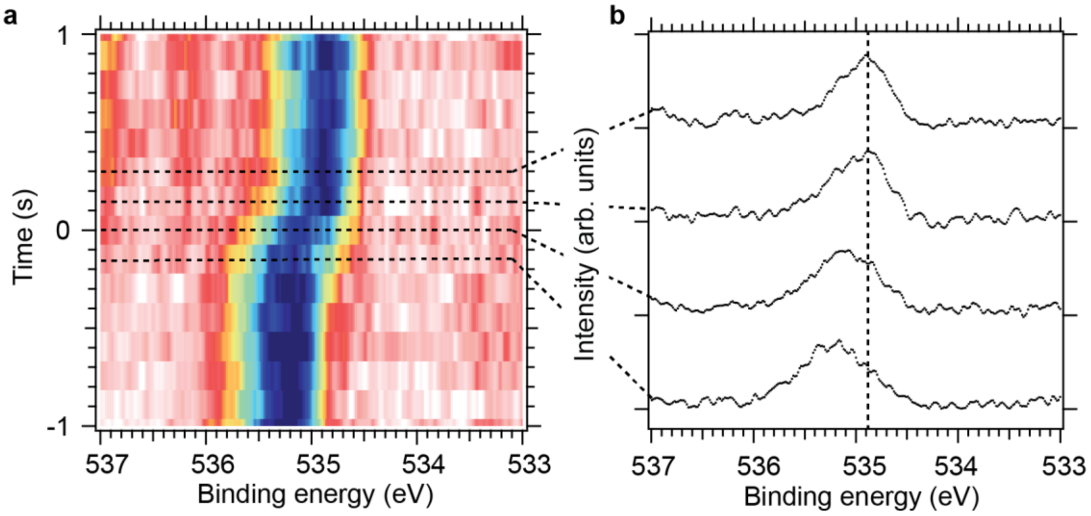

Supplementary Figure 4. **Illustration of the instantaneous work function change observed with 148 ms time-resolution. a** *O 1s* image plot of the event-averaged stamp signal corresponding to a zoom-in of figure 1c of the main article. **b** single event-averaged spectra recorded along the dotted lines panel a.

The work function transition happens between t = 0 s and t = 0.148 s. The fact that the event-averaged spectrum shows a sharp transition is clear evidence that we maintained a time-resolution close to 148 ms also after event-averaging.

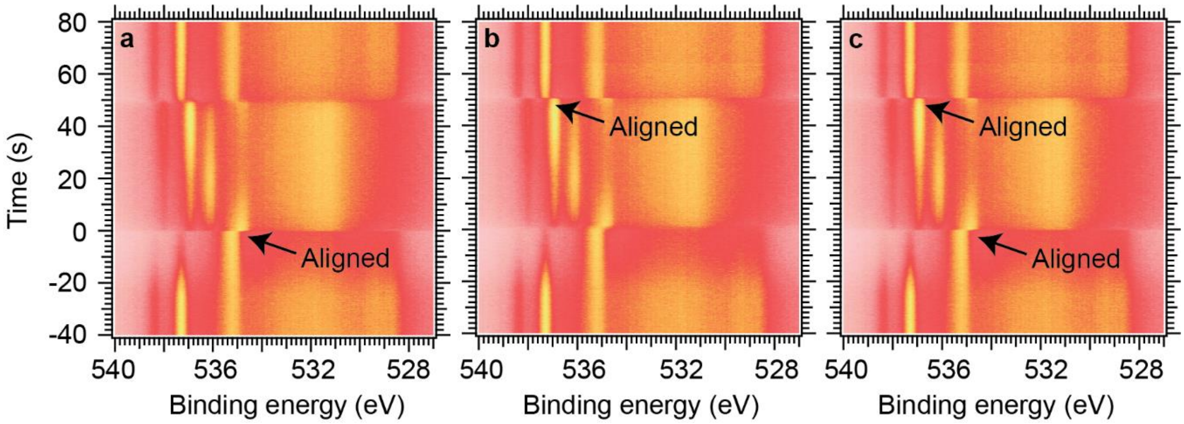

Supplementary Figure 5. **Comparison of the event-averaged raw images generated with image recognition that captures the front or end of the CO pulse or both. a** When the stamp signal at the front of the pulse indicated with a black arrow in panel a is used to generate the timing signal for formation of the event-averaged image a sharp contrast is observed at the front of the pulse (t = 0 s) both for gas phase components and surface components (compare with figure 1 for peak assignment). However, the contrast is less sharp at the end of pulse at t = 50 s. **b** The exact opposite behaviour is observed when the stamp signal at the end of the pulse is used for event-averaging. **c** demonstrates how a sharp contrast can be obtained by merging the two event-averaging images, which are averaged to the front and end of the CO pulse respectively.

This figure is one example of gas pulses not completely reproducible in terms of their arrival time. Similar effects been observed before at other beamtimes and is not surprising thinking of complex tube arrangement in the gas dosing system. Examples of this can be seen in figure 11 and 13 of ref [2].

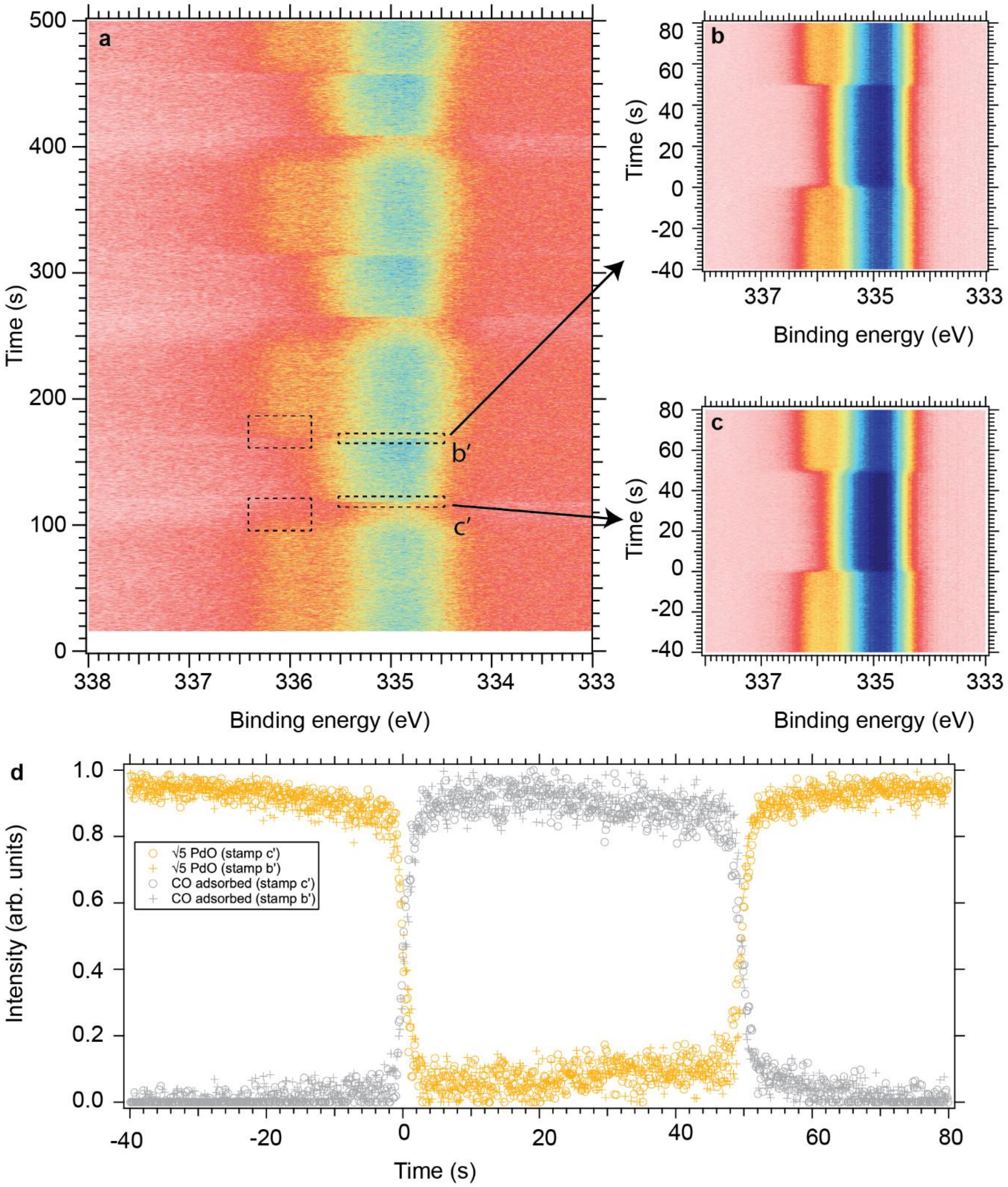

Supplementary Figure 6. **Event-averaging of time-resolved *Pd 3d*$_{5/2}$ data corresponding to figure 1 of the main article**. **a** *Pd 3d*$_{5/2}$ raw data acquired with 7 Hz and a photon energy of 410 eV. **b** Event-averaged image plots of 58 pulses (840 spectra) using the stamp signals marked with b′ in panel a. **c** Event-averaged image plots of 58 pulses (840 spectra) using the stamp signals marked with c′ in panel a. **d** Curve fitted intensities of the √5 oxide (orange circles/crosses using stamp c′ and b′, respectively) and adsorbed CO (grey circles/crosses using stamp c′ and b′, respectively) normalized to the maximum value.

To account for different electron transmission through the gas phase, the image plots in panels b and c were normalized to the total *Pd 3d*$_{5/2}$ intensity. In contrast to figure 1e and supplementary figure 7d the transition between the √5 oxide covered surface and CO covered surface is gradual taking approximately 2 s referring to panel d here. This apparent discrepancy is caused by the weak stamp signals that make it difficult to achieve a unique timing signal of the transition event on the surface. Many different time signals were tested (examples shown with dotted squares in panel a, but the quality of the data does not allow a similar quality as the *C 1s* and *O 1s* data. Supplementary movie 4 demonstrates and documents how the curve fitting of this figure was done.

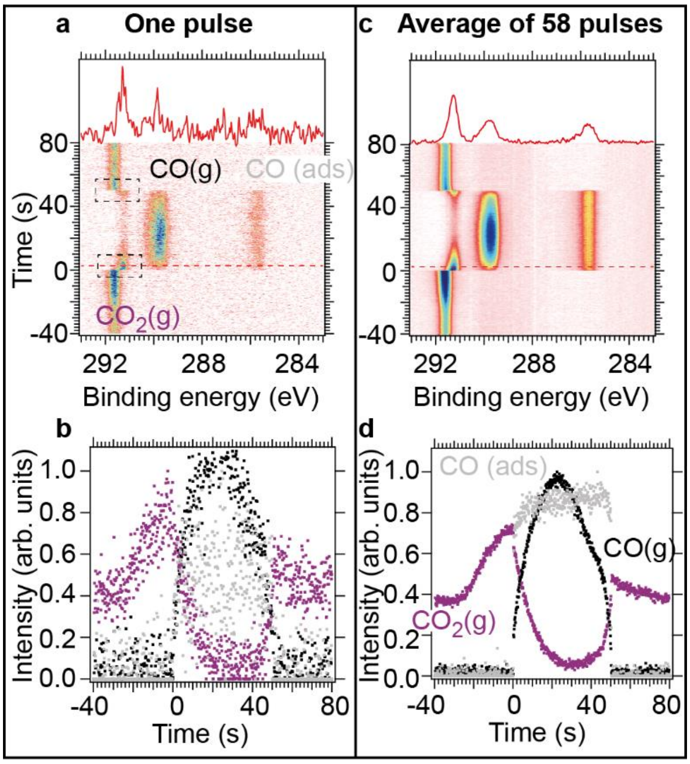

Supplementary Figure 7. **Analysis of time-resolved *C 1s* before and after event-averaging with same experimental parameters as figure 1 of the main article.** **a** and **c** show *C 1s* image plots acquired over a single pulse and averaged over 58 pulses, respectively, using a photon energy of 410 eV. At the top of both panels a single spectrum acquired along the dotted red line is shown. Spectra were recorded with 6.16 Hz and each image plot shows 739 single spectra. **b** and **d** show the relative CO and CO_2 signals and relative CO coverage derived from the image plots in panel a and b. The following colours and markers are used: CO(g) black, CO_2(g) purple, CO_{ads} light grey squares. The reason that relative concentrations rather than partial pressures are shown in the plot is that the O_2 pressure is unknown from the *C 1s* data. The curve fitting of all spectra is shown in supplementary movie 5 and 6.

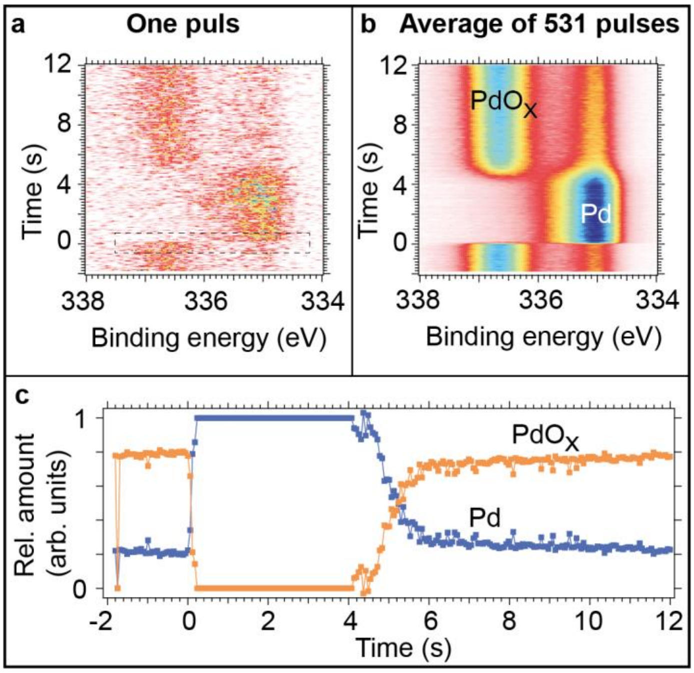

Supplementary Figure 8. **Analysis of time-resolved and event-averaged *Pd 3d*$_{5/2}$ spectra corresponding to figure 2 of the main article. a** Image plot of *Pd 3d*$_{5/2}$ spectra recorded with 17 Hz and acquired with a photon energy of 4.6 keV before event-averaging. **b** *Pd 3d*$_{5/2}$ i*mage plot* of 531 event-averaged pulses. **c** Quantitative analysis obtained by curve fitting all spectra in panel b. The following markers are used in panel c: PdO_x oxide intensity orange squares and metallic Pd blue squares. Panel a and b shows 240 spectra each. To account for different electron transmission through the gas phase caused by the He pulses, both image plots were normalized to the total *Pd 3d*$_{5/2}$ intensity. Pulsing parameters were identical to figure 2 of the main text. The curve fitting of all spectra are shown in supplementary movie 7.

The thickness of the PdO_X film is estimated to be at the order of 25 Å from the relative intensity of the PdO_X component of 0.8 shown in supplementary figure 6 using the method shown in Goodwin et al. [3]. In brief, this method incorporates the x-ray attenuation due to grazing incidence into standard XPS intensity equations. Expected XPS intensities were calculated for Pd oxide thicknesses from 0 to 100 Å, and the experimental data was then compared to the simulated results. To simulate the XPS intensity bulk densities of Pd and PdO were assumed, 12.03 and 8.3 respectively, each layer is assumed to have no roughness and no mixing. TTP-2M model was used for electron mean free path [4] and the Sergey Stepanov x-ray server [5, 6] was used to calculate x-ray field intensity within the material using the Henke model [7].

Supplementary References

[1] Hendriksen, B. L. M. *et al.* The role of steps in surface catalysis and reaction oscillations. *Nat. Chem.* **2,** 730–734 (2010)

[2] Wallander, H. Observing phase changes in real time on ultra-thin Iron oxide surfaces, Lund University, 2019. https://lup.lub.lu.se/student-papers/search/publication/8987707

[3] Goodwin, C. M. *et al.* The Structure of the Active Pd State during Catalytic Carbon Monoxide Oxidization. *J. Phys. Chem. Lett.* **12**, 4461–4465 (2021)

[4] Standard Reference Data, N. NIST Standard Reference Database 71. 1999, DOI:10.18434/T48C78.

[5] Sergey Stepanov's X-ray Server https://x-server.gmca.aps.anl.gov/ (accessed December 10, 2020)

[6] Stepanov, S. A. X-Ray Server: An Online Resource for Simulations of x-Ray Diffraction and Scattering. In Advances in Computational Methods for X-Ray and Neutron Optics; Sanchez del Rio, M., Ed.; SPIE: Denver, **5536**, 16-26 (2004)

[7] Henke, B. L., Gullikson, E. M. & Davis, J. C. X-Ray Interactions: Photoabsorption, Scattering, Transmission, and Reflection at E = 50-30, 000 EV, Z = 1-92. Atomic Data and Nuclear Data Tables **54**, 181–342 (1993)
