# Supplementary material for: Stroboscopic operando spectroscopy of the dynamics in heterogeneous catalysis by event-averaging
Source: Nat Commun. 2021 Oct 21;12:6117. doi: 10.1038/s41467-021-26372-y (PMC8531341; doi:10.1038/s41467-021-26372-y)
Supplement: Supplementary file 3 — Description of Additional Supplementary Files [file 41467_2021_26372_MOESM3_ESM.pdf]

Description of Additional Supplementary Files

File Name: Supplementary Movie 1
Description: **Illustrates the event-averaging of 10 pulses using the stamp-image shown in the dotted red box**. The *O 1s* raw data in the movie were recorded with 6.75 Hz and are shown as a running movie in the top left panel of the movie file. To reduce the file size, the movie was rendered with 8 frames per second and 20 spectra displacement between two subsequent frames. Thus, the time in the movie is accelerated with a factor of ~ 25.
The bottom panel in the movie shows the absolute difference of the stamp signal frame referenced to the value when the movie was started. In more detail, the absolute difference is calculated as the integral over the absolute difference of each pixel point and its initial value within the rectangle. In the bottom panel the integral value is plotted at the time value read by the lower edge of the dotted square in the top left panel of the movie. Once a minimum is found in the absolute difference signal corresponding to a matching image within the dotted square the entire image displayed in the top left panel is added to the top right panel. Finally, the colour scale in top right image is divided by the number of images added.

While the movie was rendered with 20 spectra displacement between two subsequent frames, the real analysis was done with single spectra displacements. The time values of each minimum in the lower panel were determined by fitting an appropriate polynomial around each minimum. This results in a very accurate determination of the time for each matching event as demonstrated by supplementary figure 4.

File Name: Supplementary Movie 2
Description: **This movie demonstrates and documents how the curve fitting of figure 1c and the integrated values shown in figure 1e have been obtained.**

File Name: Supplementary Movie 3
Description: **This movie demonstrates and documents how the curve fitting of figure 2b and the integrated values shown in figure 2e have been obtained.**

File Name: Supplementary Movie 4
Description: **This movie demonstrates and documents how the curve fitting of supplementary figure 6c and the integrated values shown in supplementary figure 6d were obtained.**

File Name: Supplementary Movie 5
Description: **This movie, demonstrates and documents how the curve fitting of *C 1s* spectra without event-averaging shown in supplementary figure 7a and the integrated values shown in panel b have been obtained.**

File Name: Supplementary Movie 6
Description: **This movie, demonstrates and documents how the curve fitting of event-averaged *C 1s* spectra shown in supplementary figure 7c and the integrated values shown in panel d have been obtained.**

File Name: Supplementary Movie 7
Description: **This movie demonstrates and documents how the curve fitting of supplementary figure 8b and the integrated values shown in supplementary figure 8c have been obtained.**
